# Complex DNA knots detected with a nanopore sensor

**DOI:** 10.1038/s41467-019-12358-4

**Published:** 2019-10-02

**Authors:** Rajesh Kumar Sharma, Ishita Agrawal, Liang Dai, Patrick S. Doyle, Slaven Garaj

**Affiliations:** 1 Department of Biomedical Engineering, National University of Singapore, 4 Engineering Drive 3, Singapore, 117583 Singapore; 2 Singapore-MIT Alliance for Research and Technology Centre, 1 CREATE Way, Singapore, 138602 Singapore; 3 Centre for Advanced 2D Materials, National University of Singapore, 6 Science Drive 2, Singapore, 117546 Singapore; 4 Department of Physics, City University of Hong Kong, 83 Tat Chee Avenue, Kowloon, Hong Kong China; 5 Department of Chemical Engineering, Massachusetts Institute of Technology, Cambridge, MA 02142 USA; 6 Department of Physics, National University of Singapore, Singapore, Science Drive 3, Singapore, 117551 Singapore

**Keywords:** Nanoscale biophysics, Single-molecule biophysics, Biological physics

## Abstract

Equilibrium knots are common in biological polymers—their prevalence, size distribution, structure, and dynamics have been extensively studied, with implications to fundamental biological processes and DNA sequencing technologies. Nanopore microscopy is a high-throughput single-molecule technique capable of detecting the shape of biopolymers, including DNA knots. Here we demonstrate nanopore sensors that map the equilibrium structure of DNA knots, without spurious knot tightening and sliding. We show the occurrence of both tight and loose knots, reconciling previous contradictory results from different experimental techniques. We evidence the occurrence of two quantitatively different modes of knot translocation through the nanopores, involving very different tension forces. With large statistics, we explore the complex knots and, for the first time, reveal the existence of rare composite knots. We use parametrized complexity, in concert with simulations, to test the theoretical assumptions of the models, further asserting the relevance of nanopores in future investigation of knots.

## Abstract (web summary)

Nanopore microscopy is capable of detecting knots in DNA polymers, a common structural feature. Here the authors show nanopore sensors can map the equilibrium structure of these knots between tight and loose states.

## Introduction

Nanopore sensors have become an important tool for investigation of biomolecules: protein pores^1^ became an indispensable tool for DNA sequencing;^2–4^ solid-state nanopores^5^ have been used for the detection of DNA and RNA conformations^6,7^ and protein fingerprinting;^8^ and nanopores in two-dimensional membranes such as graphene^9–11^ and transition metal dichalcogenides^12,13^ are being explored for high-resolution analysis of biomolecules. The nanopore detection of knots in long DNA molecules had been investigated theoretically^14–18^, and recently the first experimental observation of the knots with nanopores has been reported^19^. In this work, we demonstrate that the nanopore sensor optimally designed, could map the equilibrium configuration of the DNA knots, without nanopore-induced sliding or tightening of the knots. We use such nanopores to explore the distribution and complexity of the knots in DNA molecules with unprecedented statistics, and compare the results with numerical simulations. We demonstrate the persistence of very loose knots in DNA molecules that have eluded detection in many single-molecule experiments—such observation has implication on the understanding of the efficiency of cellular mechanism responsible for unknotting of the molecules^20^, and has technological implications on the efforts in continuous nanopore sequencing of megabase-long DNA segments^21^.

At the molecular scales, knotting is a very common phenomenon found in many molecules and processes essential to the proper functioning of life, such as DNA replication, viral DNA packaging, etc.^22–24^. The knots may affect and influence the structure and stability of DNA and proteins^20,25,26^, and a whole enzyme-based molecular machinery has evolved to control and manipulate knots^20,27^.

Several bulk and single-molecule experimental tools have been developed to create, observe and characterize knots in DNA. Gel electrophoresis, a bulk technique, has been extensively used over the past few decades to study knots but it is limited to circular DNA and small lengths of molecules up to 10 kbp^28–30^. While bulk measurements offer only ensemble-averaged properties of the knots without intricate information, the single-molecule experimental techniques—such as optical tweezers^31^, fluorescence imaging in micro-fluidic and nano-fluidic channels^32–34^, and electron and atomic force microscopies^35–37^—are limited to the investigation of small molecules and low statistics. To bridge this statistical gap, we employ nanopore microscopy, as a single-molecule technique with a comparatively high throughput, which allows us to investigate not only the dominant configurations, but also the infrequent events.

**Fig. 1:**
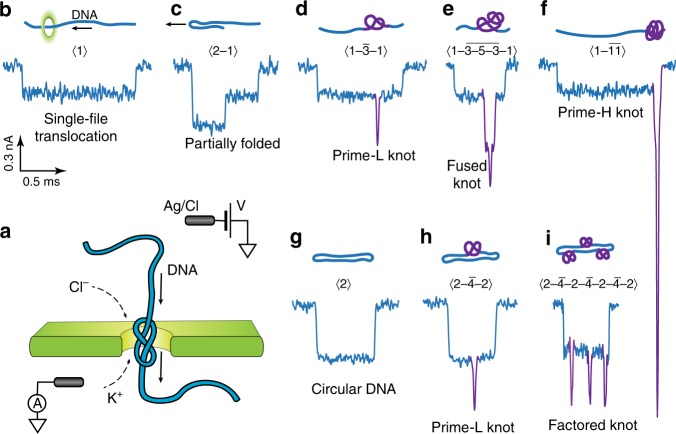
Translocation events signatures of knotted and unknotted 48.5 kbp lambda DNA molecules. **a** Schematic image of the translocation of a DNA molecule through a nanopore. **b**–**f** Examples of the current blockade signals (events) assigned to linear DNA molecules in different configurations, with corresponding sketch of DNA trajectory and SPIRaL designation. The unknotted parts of the signal and the sketch are color-coded with blue, while parts assigned to knots are presented in purple. **g**–**i** Similar signals assigned to knots on circular DNA molecules. A rare factored composite knot comprising of three factor knots is depicted in panel **i**. All events are plotted on the same scale; time-current scale is presented in the figure

In a typical nanopore experiment, a thin membrane, perforated with a single nanopore, separates two chambers with aqueous salt solution (Fig. 1a). The nanopore size is usually comparable to the sizes of the targeted biomolecules. When a voltage bias is applied across the membrane, a DNA molecule from one chamber is electrophoretically driven through the nanopore in a linear fashion (Fig. 1b). As it translocates, the molecule partially blocks the pore, leading to a transient drop in the ionic current measured through the nanopore (current blockade, *∆I*_B_). The magnitude of the blocked current is a very sensitive measure of the geometrical and physical properties of the part of the biomolecule residing within the sensing region of the nanopore at that given instance.

In this study, we use nanopores to explore statistically relevant sample of DNA knots. Firstly, in order to understand the complex conformation space of the knotted events, we introduce a classification scheme (SPIRaL classification) for the nanopore signals, and map it to Alexander–Briggs notation^38^. By carefully investigating the individual knotted events and their statistical distribution, we demonstrate that the knots, for well-chosen experimental parameters, do not experience noticeable tightening or sliding during the translocation event. Our translocation events reflect the instantaneous configuration of the knot in the solution, at the moment preceding the capture of the DNA molecule by nanopore.

Secondly, we investigate the rare complex knots and the frequency of their occurrence. We compare the experiments with numerical simulations, and use the knot complexity parameter to test the general theoretical assumptions of the polymer model.

Next, we experimentally validate the two distinctive modes of knot translocation through nanopores that involve widely different tension forces on the knots, which was recently proposed by a simulation study^18^.

Finally, we shed more light on a puzzle of the size range of the equilibrium knots. The previous nanopore experiments reported observation of only tight knots^19^ (less than 300 nm of DNA contour), which is likely due to the nanopore-induced tightening of the knots. Some experimental techniques reveal only tight knots^31^, while others report loose knots^39–41^. In our experiments, with large statistics and lack of the spurious knot tightening, we observe the co-existence of both tight (few tens of nanometers) and loose knots (few micrometers in contour length), indicating that the inconsistencies in previous reports could be attributed to the selection bias of different experimental techniques.

## Results

### Individual knot events

We investigated equilibrium knots in 48.5 kbp long linear and circular lambda DNA molecules by translocating them through a nanopore of diameter, *D* = 20 nm, fabricated in a thin (*h* = 20 nm) silicon nitride membrane in 1.5 M buffered KCl solution at 250 mV driving voltage. We observed 4348 translocation events, after filtering out short bumps and fragments. For each event, we analyzed individual configuration of the DNA molecule based on its ionic current signal during the translocation event. At high ionic strength, the current blockade $\Delta I_{\mathrm{B}}\left( t \right)$ is proportional number of DNA filament traversing the pore at that instance. Fully extended DNA molecule, translocating in the single file confirmation (Fig. 1b), would give rise to a constant current drop, $\Delta I_{\mathrm{B}}=I_0$, whereas folded DNA or circular DNA molecules would give a drop of $\Delta I_{\mathrm{B}} = 2I_0$. The step size, $I_0\;=\;0.23\;\pm\;0.04\;{\mathrm{nA}}$, corresponds to the current blockade associated with a single filament of the double-stranded DNA molecule blocking the pore. Figure 1b, c and g show the current traces of unfolded linear, folded linear, and circular DNA molecules, respectively.

The signature of a knot is a higher-order stepwise drop current blockade, or series of *i* steps, with a magnitude of

1 $$\Delta I_{{\mathrm{knot}}}^{({\mathrm{i}})}\;=\;kI_0\;=\;\left( {n\;+\;l} \right)\;I_0$$

where *n* = 1,2 is the number of un-knotted DNA strands passing through the pore, and *l* is additional current drop due to knots. It should be noted that knots should have $l\;\ge\;2$. Current blockade signals assigned to knots in different configurations are shown in Fig. 1.

We reason that the ionic current signal from a knotted molecule is a good measure of the equilibrium properties of the knot in solution. Firstly, the polymer relaxation time (Zimm time) is much larger than the translocation time, preventing the yet-not-translocated part of the DNA molecule to change configuration during the translocation. Secondly, although each individual DNA molecule experiences velocity fluctuations during translocation^42,43^, the constant average velocity approximation for calculating knot position is still valid when applied to statistically large sample of molecules. Finally, we will present evidence below that nanopores, in our experimental configuration, do not induce any noticeable knot sliding or tightening. We postulate that, due to all those effects, 3-dimensional knot transforms into a topologically equivalent quasi-1D form of the molecule as it passes through the pore, and it retains all the relevant geometrical parameters of the initial, equilibrium 3D knot^18^.

### Statistical analysis of knots

Statistical analysis of the knotted DNA events allowed us to overcome the inherent experimental limitations associated with the individual events, and to gain a broader picture of the structure and dynamics of the knots. To facilitate the analysis, each of the complex translocation events was reduced to a set of numerical identifiers appropriate for a given analysis.

For a general DNA translocation event, the primary physical quantifiers are the total translocation time, *T*_R_, and the average current blockade $\Delta I_{{\mathrm{avg}}}$. The event charge deficit is defined as the total charge blocked from the passage of the molecule through the nanopore during the translocation event:

2 $${\mathrm{ecd}} = \mathop {\smallint }\limits_0^{T_{\mathrm{R}}} \Delta I_{\mathrm{B}}\left( t \right){\mathrm{d}}t\;=\;\Delta I_{{\mathrm{avg}}} \;\cdot\; T_{\mathrm{R}}$$

**Fig. 2:**
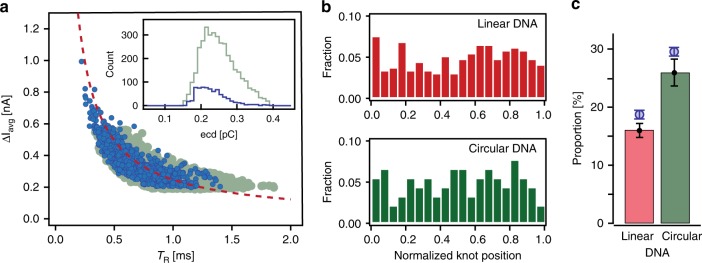
Detection of equilibrium knots. **a** Scatter plot of average current blockade $\left( {\Delta I_{{\mathrm{avg}}}} \right)$ versus translocation time (*T*_R_) for unknotted (light green circles) and knotted events (darker blue circles). All the events follow the hyperbolic curve of constant event charge deficit (ecd), i.e., constant apparent length of the molecule. Inset: histogram of ecd for knotted events (lighter green) and unknotted events (darker blue) shows similar average values (0.25 pC for unknotted and 0.23 pC for knotted events) and distributions, indicating no effects of knots on the DNA translocation speed. **b** Histogram for normalized center position of knots on linear (red), as well as circular (green) DNA molecules shows that the knots are randomly located along the length of the molecule. **c** Proportion of lambda DNA molecules with knots, in linear and circular configurations; experiment results presented with bars and simulation with circles (effective chain width *w*_DNA_ = 2.4 nm). Error bars show the statistical error, assuming Poisson statistics. Source Data are provided as a Source Data file

Plotting $\Delta I_{{\mathrm{avg}}}$ vs. *T*_R_ in Fig. 2a, we observed that both unknotted and knotted events distribute along the same hyperbolic fit of constant ecd. The inset of Fig. 2a shows the distribution of ecd for events scored as knotted and unknotted, with very similar average values (0.25 pC for unknotted and 0.23 pC for knotted events) and distributions. Hence, we postulate that: (a) all DNAs passed through the pore freely, with no (stick-slip) interaction with the nanopore walls; (b) the knots do not slow down the translocation of DNA. The observed spread in the ecd distributions is due to the statistical variation of the drag forces experienced by different initial conformations of the DNA molecules^42^.

Next, we calculated the relative position of a knot along the translocating DNA molecule, $x=t_{{\mathrm{knot}}}{\mathrm{/}}T_{\mathrm{R}}$, where *t*_knot_ is the time between start of the DNA translocation and the mid-point of the knot peak. We observed that the position of the knot is equally distributed along the length of both linear and circular DNA molecules (Fig. 2b). This suggests that, for our experimental parameters, we did not observe sliding or slipping off of the knots along the DNA. It is worth noting that a previous report^19^ observed knot sliding for all voltages except for the smallest of the voltages. We attribute the difference to the difference in the geometry, chemical environment and the translocation timescales between the experiments.

The other useful statistical quantifiers we use are the size of the knots, complexity of the knots as identified by the maximum current drop, and the configuration of the knots during translocation. In order to further expand the statistical analysis, we defined a classification scheme for the nanopore signals arising from knots.

### Classification of knots

A convenient nomenclature was assigned (SPIRaL classification) to categorize nanopore signals from a variety of DNA knot configurations (Fig. 1b–i) and to facilitate further discussion. We assigned a digital signature to each current blockade event of the form $\langle n_1-\bar k-n_2\rangle$, where *n*_1_ and *n*_2_ are, respectively, the numbers of DNA strands inside the nanopore just before and after the knot. Akin to the previous definition, $\bar k$ is the number of strands associated with the maximum current drop $\Delta I_{{\mathrm{max}}}$ within the knot portion of the current blockade. The *k*-number is an indicator of the topological properties of the knot, as it represents the number of strands passing concurrently through the pore. In this classification, 〈1〉 represents a single-file or an unfolded translocation (Fig. 1b), 〈2〉 represents either a circular or a fully folded molecule (Fig. 1g), and 〈2-1〉 is a partially folded molecule (Fig. 1c), entering the nanopore with a folded loop. A simple trefoil knot 3_1_ on a single-file DNA molecule would be represented by $\langle 1-\bar 3-1\rangle$ (Fig. 1d), while the same knot on a circular molecule would be represented by $\langle 2-\bar 4-2 \rangle$ (Fig. 1h). The numbers with an overline represent the knotted section, as compared to the extended DNA or folds in the DNA molecule, which are represented by regular numbers. It is noteworthy that once the DNA molecule enters the nanopore, a combination of the strong electrophoretic and drag forces straighten any nugatory folds, leaving topological knots clearly visible^44–49^.

Partial signal degeneracy is an inherent limitation of the nanopore experiments, where a current-blockade signature could result from different knot topologies. For example, 3_1_ and 4_1_ knots would give rise to the same $\langle 1 - \bar 3 - 1\rangle$ classified events. While the degeneracy in nanopore mapping prevents the complete topological identification of each knot, we could still gain significant insight into the physics of polymer knotting by employing statistical analysis of the events within the SPIRaL phenomenological classification. To that end, we classified all the events into lower-order prime knots (“prime-L”), higher-order prime knots (“prime-H”), and composite knots (see Fig. 1 and Supplementary Table 1).

By definition, prime knots are those whose only factors are themselves and unknot, while composite knots are comprised of multiple factor knots. Prime-L knots are defined to correspond to events with *l* = 2, for example $\langle 1-\bar3-1\rangle$, and to the most frequent trefoil 3_1_, quatrefoil 4_1_, cinquefoil 5_1_ knots (Fig. 1d, h). Prime-H knots have $l\;\ge\;4$, and correspond to knots with larger number of crossings, such as 6_2_, 6_3_, etc (Fig. 1f). This separation of prime knots into lower and higher order categories is valid in most of the cases, except for the twisted conformation of each higher order prime knot (7_1_, 8_1_, … *F*_1_), which can have a *k*-number equal to 3. However, they are increasingly rare, and do not affect our conclusions in any meaningful sense. Since the conformation $\langle 1-\bar4-1 \rangle$ (*l* = 3) is physically impossible, the prime-H knots category is well separated, non-degenerate, representing exclusively the prime knots with more than four crossings.

Composite knots are combinations of various prime knots that can be factored into their components, and are commonly represented as $F_1\# F_2\# \ldots \# F_{\mathrm{i}}$, where *F*_1_, *F*_2_, and *F*_i_ are factor knots. In the nanopore experiments, the composite knots would be represented with a series of deep current-blockade steps (see Fig. 1e, i). However, not all composite knots are clearly resolvable with nanopores due to the limited temporal resolution. Those that have factors well-separated from each other $\left({\gtrsim200\;{\mathrm{nm}}}\right)$ along the DNA strand are clearly resolvable and are called factored composite knots (Fig. 1i), whereas others that have factors too close to each other are observed as fused signatures of composite knots. These fused composite knots (Fig. 1e) have multiple sub-levels within the knot current peak, wherein each level refers to a factor knot. A second possibility, though less likely, is that the fused composite knots represent intertwined factored knots, a form whose existence is predicted in literature^50,51^. The signal in Fig. 1e could correspond to either a fused or intertwined knot. In line with the previous definition, we represent factored composite knots as $\left\langle {n_1-\overline {k_1}-n_2-\overline {k_2}-n_3 \ldots-\overline {k_i}-n_{i\;+\;1}} \right\rangle$, while the fused composite knots are represented as $\left\langle {n_1-\overline {k_1-k_2{\mathrm{-\ldots }}k_i}-n_2} \right\rangle$.

Supplementary Table 1 gives an overview of the SPIRaL classification scheme. The probabilities of occurrence of each knot type described above, example events, and a non-exhaustive list of examples mapped to the Alexander-Briggs notation^38^ for knots, are presented in the table.

### Complexity of the knots

**Fig. 3:**
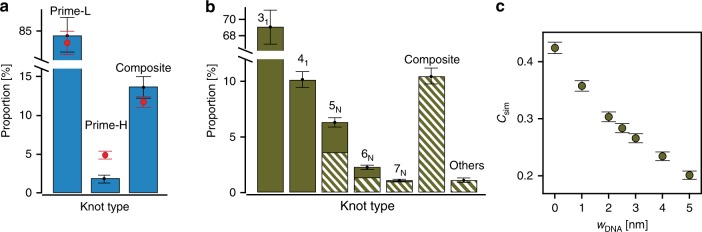
Percentages of various knot types and the complexity of knotting. **a** Histogram of experimental (blue bars) and simulated (red crosses) knotting probabilities of lower order prime (prime-L) knots, higher order prime (prime-H) knots, and composite knots. **b** Proportion of different types of knots in a simulated lambda DNA (width, *w*_DNA_= 2.5 nm). Solid bars represent lower order prime (prime-L) knots while the partially filled bars represent complex (prime-H and composite) knots. **c** Plot of simulated knotting complexity (*C*_sim_) of lambda DNA, as a function of effective DNA width (*w*_DNA_) indicates the strong dependence of the occurrence of complex knots on the effective diameter of DNA. All error bars show the statistical error, assuming Poisson statistics. Source Data are provided as a Source Data file

Figure 3a depicts the proportion of detected knots for each defined category. The knots categorized as prime-L, prime-H, or composite pose different biological challenges, and their prevalence is an important parameter when trying to understand the role of knots in biological processes^52^. Comparing the categorized events with theoretical simulations, we could gain insights into the relevant parameters controlling DNA knotting.

We performed numerical simulations using the Monte Carlo method for circular chains, and the modified PERM (Pruned-enriched Rosenbluth method) algorithm for linear chains (Details in Methods section). We simulated the ensembles of DNA conformations while varying the effective chain width *w*_DNA_ from 0 nm to 5 nm. We calculated the Alexander polynomial for each chain to determine its topology and distinguish between prime and composite knots. The simulated frequency of occurrence for different knot topologies in linear chains, for the case of *w*_DNA_ = 2.5 nm that approximately corresponds to the effective diameter of DNA for our experimental conditions, is shown in Fig. 3b. As a point of comparison between the theory and experiments, we calculated the total knotting probability $P\;=\;N_\kappa/N_{{\mathrm{total}}}$, where *N*_total_ and $N_\kappa$ denote the total number of molecules and the number of knotted molecules, respectively.

With an increase in the effective diameter of DNA (taking into account both the bare DNA diameter and the screened electrostatic repulsion between negative charges on DNA), the simulated knotting probability *P*_sim_ decreases (Supplementary Fig. 1). The knotting probability *P* in linear DNA chains shows a good agreement between experiments (*P*_exp_ = 16%) and simulation (*P*_sim_ = 18.8%) for lambda DNA of width *w*_DNA_ = 2.5 nm. We also found good agreement between experiments (*P*_exp_ = 25.9%) and simulations (*P*_sim_ = 29.6%) for circular DNA chains. In addition, by comparing the experimental and simulated knotting probabilities for linear and circular chains, we showed that the equilibrium knotting probability of circular DNA is inherently 50–65% higher than that of linear DNA (Fig. 2c).

We defined the knotting complexity as a proportion of complex knots (prime-H and composite) over the total number of knots $C\;=\;1\;-\;N_{{\mathrm{pL}}}/N_{\mathrm{\kappa}}$, where *N*_pL_ is number of prime-L knots. The theoretical number of prime-L knots *N*_pL_ is summation of the numbers of 3_1_, 4_1_, 5_1_, 6_1_, 7_1_, and 8_1_ knots. The knotting complexity should depend on the effective diameter of the molecule and it is a useful parameter for evaluating the simulation model. We found that the observed *C*_exp_ = 15.6% is rather close to the values predicted for *w*_DNA_ = 2.5 nm by our theoretical model *C*_sim_ = 16.6%, supporting the model in our simulations.

Using traditional single-molecule techniques (e.g., optical, electron and atomic force microscopies), it is hard to observe composite knots, and nearly impossible to deduce any statistically significant conclusions. With high temporal and spatial resolution, nanopores enable clear observation of composite knots with multiple factor knots, in statistically significant numbers. For example, Fig. 1i shows a rare triple factored composite knot. Although overlooked in the previous nanopore study^19^, we observed the proportion of composite knots as large as 13.7%, out of which 2.6% are clearly resolved factored knots. Such large proportion of composite knots matches well with the value of 11.7% from our simulations for *w*_DNA_ = 2.5 nm (Fig. 3a), and indicates that the composite knots could play a significant role in key biological processes such as viral DNA packaging^23^, protein folding^53^, and intracellular catalysis^54^.

### Modes of knot translocation through nanopores

A recent theoretical study^18^ identified two quantitatively different modes of the knot translocation in circular DNA molecules, where knots are subjected to different stretching forces. Careful experimental investigation of those modes enabled us to determine if knots tighten during the translocation.

**Fig. 4:**
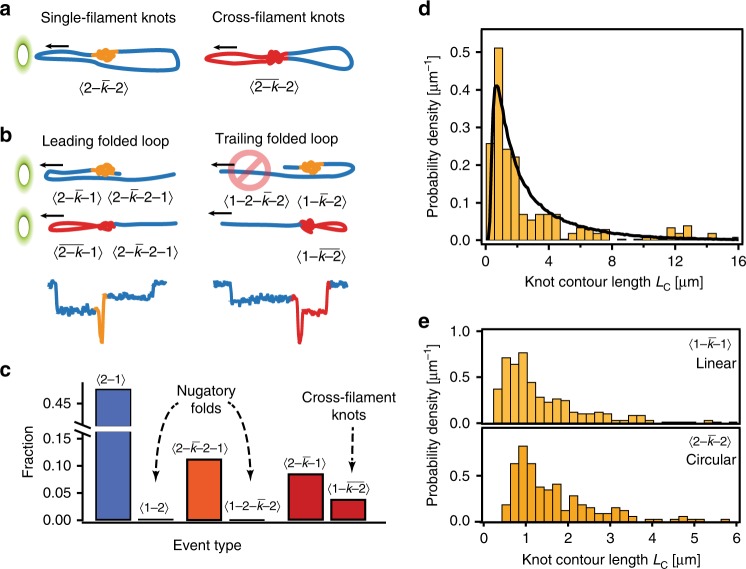
Two modes of DNA knot translocation and the size distribution of knots. **a** Distinctive modes of knot translocation in circular DNA; a single-filament knot resides on one strand of the translocating DNA, cross-filament knot straddles over two translocating filaments, experiencing significantly lower tension force. **b** Single-filament and cross-filament knots in translocating linear DNAs with leading (left) and trailing folded loops (right). **c** Proportions of knotted events in different configurations, where high relative prevalence of $\langle 1 - \overline {k{\mathrm{ - 2}}}\rangle$ knots demonstrates the existence of cross-filament mode of translocation. **d** Histogram of the measured knot contour lengths shows coexistence of both tight and loose knots. Black curve represents simulated distribution of knot sizes for DNA population with *w*_DNA_ = 2.5 nm. **e** Histograms of knot sizes for linear DNA (top) and circular DNA (bottom) shows very similar distribution, despite different modes of translocation, indicating lack of knot-tightening in our experiments. Source Data are provided as a Source Data file

In the first mode, a knot resides on only one of the two translocating filaments of the circular DNA molecule (single-filament knots)—see Fig. 4a. In this case, the tension propagates along the knotted filament from the pore to the knot, and could lead to the tightening of the knot. The same forces would act on any knots on a linear DNA translocating in a single-file, i.e., $\langle 1-\bar k-1\rangle$.

In the second mode of translocation, the knot straddles two filaments during translocation (cross-filament knots), which removes the net tightening force on the knot. The simulation study^18^ reported that about 50% of the knotted circular molecules translocate in the cross-filament mode, and the authors predict significant reduction in propensity for knot tightening in that case.

In circular DNA, it is impossible to experimentally distinguish between the two modes of translocation, as they would lead to the same current-blockade signatures. Here we show that inspection of partially folded linear DNA molecules could distinguish between the two modes of the translocation, at least in some cases (Fig. 4b, c).

The folded translocations with leading double strand 〈2-1〉 are rather common, and we observed 2079 such events out of 4348 observed translocations (47.8%). In those cases, the DNA molecule is captured at a point along the molecule and pulled in the folded form through the nanopore. On the other hand, the folded translocations with a leading single strand 〈2-1〉 are highly unlikely, due to the tension propagation along the leading strand that would straighten out the rest of the DNA molecule. Indeed, we observed only 6 such events out of 4348 events (0.14%).

Inspecting knots on folded parts of linear molecules, we observed 62 $\langle \overline {2-k}-1\rangle$ and 83 $\langle 2-\bar k{\mathrm{-2-}}1\rangle$ events, where a knot resided on a leading double strand (out of 739 total knots). In those cases, we could not distinguish between single-filament knots and cross-filament knots. Surprisingly, we observed 28 $\langle 1-\overline {k{\mathrm{-2}}}\rangle$ events. Such translocating configuration could only exist if two criteria are met: the knot straddles the two filaments; and it acts as a strong anchor point that pulls the whole loop into the nanopore, preventing any sliding or tightening of the knot. In contrast, a single-filament knot would not be able to reduce the tension propagation along the molecule and would translocate exclusively in $\langle 1 - \bar k - 1\rangle$ configuration. Figure 4c displays the event statistics. The anchor concept is also supported by the complete lack of any $\langle 1{\mathrm{ - 2 - }}\bar k - 2\rangle$ events, although we would expect them to be much more frequent than $\langle 1 - \bar k - 2 \rangle$ events in the absence of the tension forces.

Our measurements of the total number of the cross-filament knots represents only a lower bound, as we could not distinguish between cross-filament and single-filament knots in 146 events with a leading double strand ($\langle \overline {2 - k} - 1\rangle$ and $\langle 2 - \bar k{\mathrm{ - 2 - }}1 \rangle$), We note that in the case of the cross-filament knots, the big loop of the folded strand is a part of the knot, and should be counted towards the total contour length of the knot.

### Knot sizes

The size of knots in biological molecules could determine the dynamics of crucial biological processes. For example, type IIA DNA topoisomerase actively unknots DNA molecules, but it works efficiently only with very tight knots^55^. Understanding the range of knot sizes of the equilibrated DNA molecules would reveal if additional mechanisms should be invoked to retain the low occurrence of knots in vivo^20^. Also, while research on the sizes of polymer knots has been going on for many decades, a direct comparison of DNA knot sizes deduced from theory^56^, simulation^57^, and experiment^19^ was only recently made.

There is not consensus in literature regarding the distribution of knot sizes in DNA molecules—the measured contour length of the knots varies significantly between reports using different measurement techniques. Tight knots were observed with optical tweezers^31^, having length in the range of *L*_C_ = 200–500 nm, and with early nanopore experiments^19^ in the range of *L*_C_ < 300 nm; much larger knots^39–41^ in the range of *L*_C_ = 1.5–3 μm have been observed in micro-channels and nano-channels. Such wide variation in reported knot sizes could arise from the selection biases of the experimental techniques, tightening of the knots due to an applied force, or lack of sufficient statistics.

Our nanopore experiments allow us to accurately map the size distribution of DNA knots due to the large number of events sampled. We calculated the knot contour length *L*_C_ by comparing the event charge deficit of knot (ecd_knot_) to that of the entire molecule (ecd_DNA_):

3 $$L_{\mathrm{C}}\;=\;\frac{{{\mathrm{ecd}}_{{\mathrm{knot}}}}}{{{\mathrm{ecd}}_{{\mathrm{DNA}}}}}L_{{\mathrm{DNA}}}$$

where *L*_DNA_ = 16.5 μm is length of the DNA molecule. The ecd_knot_ is proportional to the number of base pairs contained within the knot, and is directly proportional to the contour length of the knots^58–61^.

A histogram of the measured knot contour lengths is depicted in Fig. 4d. We observe a peaked distribution of contour lengths with maximum probability at $L_{\mathrm{C}}^{({\mathrm{max}})} \approx 750\;{\mathrm{nm}}$, an initial exponential-like drop-off with increased length of the knots, and a constant non-vanishing tail for lengths above *L*_C_ > 5 μm. The average measured length of the knots is $L_{\mathrm{C}}^{({\mathrm{avg}})}\;=\;3.1\;{\mathrm{\mu m}}$.

Using our theoretical model, we simulated the distribution of the length for results for effective DNA width *w* = 2.5nm (overlaid with experimental data in Fig. 4d). The calculated distribution matches well with the experiments—having the most probable length of $L_{\mathrm{C}}^{({\mathrm{max}})}\;\approx\;620\;{\mathrm{nm}}$ and average value of $L_{\mathrm{C}}^{({\mathrm{avg}})}\;=\;2.9\;{\mathrm{\mu m}}$—with one notable discrepancy. Whereas the simulations predict exponential drop in the frequency as the knots become larger, we persistently observe loose knots larger than 5 μm. Though these loose knot events are present in statistically significant numbers, they comprise only a small proportion of the total knot population.

Our results on knot contour distributions differ from the prior study of nanopore detection of knots^19^ wherein the authors reported very tight knots, a majority of which had sizes smaller than 100 nm (see Supplementary Fig. 2 for comparison). The difference in their results from ours is likely due to knot sliding and tightening in their experimental circumstances—an effect demonstrated by the authors themselves^19^. Our experiments differ in the details of the choice of electrolyte and the associated timescales of the translocations. Compared to their experiments in LiCl, our experiments in KCl resulted into a 5–10 times faster translocation, which has also been observed previously^19,62^. This, in turn, resulted into a quick passage of the knots through the nanopore by allowing little time for a knot to slide at the pore entrance and get tightened. Moreover, the subtle differences in the nanopore geometries and chemistry could also affect the translocation process.

To further dismiss any knot-tightening effects in our experiments, we compared the sizes of knots in circular DNA molecules $\langle 2-\bar k-2\rangle$, and linear DNA molecules translocating in single-file configurations $\langle 1-\bar k-1\rangle$), as shown in Fig. 4e. For the $\langle 1-\bar k-1 \rangle$ events, the knots are experiencing tension forces, and could experience knot tightening. For the $\langle 2-\bar k-2\rangle$ events, the knots could translocate in either single-filament configuration or cross-filament configuration in roughly equal proportion (see Fig. 4a). In the first case, the knot experiences roughly equal forces as $\langle 1-\bar k-1\rangle$ knots, whereas in the cross-filament configuration, the knot experiences significantly smaller forces and should not be subjected to notable tightening^18^. We see that the knot size distribution in linear and circular DNA molecules does not differ significantly, supporting our assertion that knot tightening is not significant in our experiments. This observation, combined with the distribution of the knot positions, sizes, and comparison with theoretical simulations, leads us to conclude that the knots observed in our nanopore experiments effectively map the equilibrium knot distribution of free DNA molecules in the solution.

## Discussion

We demonstrated the use of nanopores for investigating the complex conformation space of DNA knots. While discussing the inherent limitations of nanopore-based detection of knots, we show how statistical analysis can be implemented to overcome these limitations and gain relevant insights about the complex knotting and knot translocation phenomena. We presented a convenient classification scheme for nanopore-based knot signatures that can be mapped to the pre-existing Alexander-Briggs classification. Through the analysis of position and size distributions, and by comparison with our simulated knotting probabilities, we showed that we were able to map the equilibrium ensemble of DNA knots using nanopores. We determined the translocation modes of knots in nanopores and also the equilibrium sizes of knots. Our analysis shows that equilibrium knot sizes range from a few tens of nanometers to a few microns, demonstrating that knots can be tight or loose in equilibrium. We also improved upon the traditional single-molecule techniques by demonstrating the clear detection of composite knots with multiple factor knots in statistically significant numbers with high resolution.

Accurate genome sequencing using nanopores depends on threading through an untangled strand of the DNA, and the venture into ultra-long reads^21^ (>100,000 bases) significantly increases the chances of interference by knots. Furthermore, the efficiency of the DNA unknotting enzymes—a possible solution to the problem—depends on the degree of knot tightness^20^. This makes the detailed understanding of the prevalence and physical characteristics of the knots not only a scientific question, but one of significant technological impact. In this work, we have demonstrated that solid-state nanopores are a critical tool to investigate the properties of equilibrium knots on single DNA molecules in solution, and could give insights into the knot structure, knotting mechanism, and its dynamics.

## Methods

### Nanopore fabrication

LPCVD grown 300-nm thick low stress silicon nitride on 525-μm thick silicon wafers were purchased from Cornell NanoScale Science and Technology Facility, Ithaca, NY, USA. Windows of free-standing silicon nitride membranes were fabricated using standard photolithography and deep reactive ion etching of a wafer, followed by the anisotropic wet etching of silicon in 33% KOH solution.

Wafers were further patterned using electron beam lithography to fabricate one mini-membrane of diameter 500 nm in each freestanding silicon nitride membrane. Deep reactive ion etching was used to thin the membrane further down to a thickness of 20 nm. The whole wafer was diced to segregate individual chips. Each chip was drilled using a focused electron beam in a JEOL 2010F electron microscope operating at 200 kV.

### Cleaning and assembly of nanopores

Nanopores were treated with acid piranha (7:3::H_2_SO_4_:H_2_O_2_) for 30 minutes and then rinsed with ultrapure water 4–5 times, followed by blow drying with nitrogen gas. Each membrane with a nanopore was placed in between two fluidic compartments filled with 1.5 M potassium chloride (KCl) buffered with 10 mM Tris and 1 mM ethylenediaminetetraaceticacid (EDTA), pH = 8.2, in such a way that the nanopore remained the only connection between the two compartments. Two freshly regenerated Ag/AgCl electrodes were connected to the compartments for ionic conductivity measurements through the nanopore.

### DNA sample preparation

Double stranded 48.5 kbp long lambda phage DNA was bought from New England Biolabs (MA, USA). DNA samples were diluted to 5 μg/mL for proper dispersion of DNA and to virtually avoid any co-translocation events, and heated to 65 °C for 5 min in 1.5 M KCl and then rapidly cooled down on ice before use in order to form mixed populations of linear and circular DNA molecules^19,63^.

### Data recoding and analysis

Data was recorded using Axopatch 200B amplifier, Digidata 1440B data acquisition system and pClamp software from Molecular Devices, LLC, CA, USA at 250 mV driving voltage and 50 kHz filter bandwidth. Data was analyzed using MATLAB scripts of the transalyzer package^64^ and some other custom MATLAB scripts.

### Simulations

To obtain the knotting probability and the knot types of DNA, we performed simulations to sample the conformations of semiflexible chains and analyzed the topology of the chain conformations. For a circular chain, we used Monte Carlo simulation to sample chain conformations. For an open linear chain, we used the modified PERM (Pruned-enriched Rosenbluth method) algorithm^57^. The polymer chain was modeled as a string of touching beads. The diameter of each bead equals the effective chain width *w*, and the contour length *L* is thus *L* = *Nw*, where *N* is the number of beads. There are only two interactions between beads: the pairwise hardcore repulsion between beads and the bending energy $E_{{\mathrm{bend}}}(\theta )/k_{\mathrm{B}}T\;=\;\left( {1/2} \right)\left( {L_{\mathrm{p}}/w} \right)\theta ^2$, with bending angles *θ*, to reproduce the persistence length *L*_p_. We set the effective DNA diameter as *w* = 2.5nm and the persistence length as *L*_p_ = 50 nm. For λ-DNA, we set the number of beads as *N* = 6596. We calculated the Alexander polynomial to determine the topology of a chain. To determine the topology of an open chain, the chain must first be closed. Here, we employed the minimally interfering closure scheme^65^ to close an open chain.

## Supplementary information

Supplementary Information

Source Data

## Data Availability

All relevant data are available from the corresponding authors on reasonable request. The source data underlying Figs. 2a–c, 3a–c, 4c–e, and Supplementary Figs. 1–4 are provided as a Source Data file.

## Code availability

All relevant code used for simulations is available from the corresponding authors on reasonable request.
