# Supplementary material for: Complex DNA knots detected with a nanopore sensor
Source: Nat Commun. 2019 Oct 2;10:4473. doi: 10.1038/s41467-019-12358-4 (PMC6775256; doi:10.1038/s41467-019-12358-4)
Supplement: Supplementary file 1 — Supplementary Information [file 41467_2019_12358_MOESM1_ESM.pdf]

Supplementary Information

Complex DNA knots detected with a nanopore sensor

Rajesh Kumar Sharma *et al.*

Supplementary Tables

Supplementary Table 1 Summary of the SPIRaL classification for knotted events observed using nanopores. All knotted events are categorized into prime-L, prime-H, factored composite and fused composite knot categories. Generalized forms of SPIRaL signatures and some examples for each category are shown, along with their probability of occurrence. Example events and physical conformations are referred to the Fig. 1 in main text of the article. Each knot category has many permutations of possible knot types (as per the classic Alexander-Briggs classification), some of which are shown as examples. Total number of knotted events $N_{\text{tot}} = 739$.

**Knot category**	**SPIRaL Knot Signature (Most frequent examples)** ***n*** **= 1 or 2** ***k*** **= 3, 4, 5,..., n**	**Occurrence (%)**	**Event Trajectory and Physical Conformation (Some Examples)**	**Possible knot types (non-exhaustive list)**
**Prime-L**	$\langle n_1\text{-}\overline{k}\text{-}n_2\rangle$<br>$\langle 1\text{-}\overline{3}\text{-}1\rangle$<br>$\langle 2\text{-}\overline{4}\text{-}2\rangle$<br>$\langle 1\text{-}\overline{4}\text{-}2\rangle$<br>$\langle 2\text{-}\overline{4}\text{-}1\rangle$	84.4±3.0	Fig. 1d	3_1, 4_1, etc
**Prime-H**	$\langle n_1\text{-}\overline{k}\text{-}n_2\rangle$<br>$\langle 1\text{-}\overline{7}\text{-}1\rangle$<br>$\langle 2\text{-}\overline{11}\text{-}1\rangle$<br>$\langle 2\text{-}\overline{14}\text{-}2\rangle$	1.9±0.5	Fig. 1f	9_1, 10_1, etc
**Composite (Factored)**	$\langle n_1\text{-}\overline{k_1}\text{-}n_2\text{-}\overline{k_2}\text{-}n_3 \ldots \text{-}\overline{k_\iota}\text{-}n_{i+1}\rangle$<br>$\langle 1\text{-}\overline{3}\text{-}1\text{-}\overline{3}\text{-}1\rangle$<br>$\langle 2\text{-}\overline{4}\text{-}2\text{-}\overline{4}\text{-}2\rangle$	2.6±0.6	Fig. 1i	$3_1\#4_1$, $3_1\#3_1$, $3_1\#3_1\#\,3_1$, etc
**Composite (Fused or Intertwined)**	$\langle n_1\text{-}\overline{k_1\text{-}k_2\text{-}\ldots k_\iota}\text{-}n_2\rangle$<br>$\langle 1\text{-}\overline{3\text{-}5\text{-}3}\text{-}1\rangle$	11.1±0.8	Fig. 1e	$3_1\#4_1$, etc

Supplementary Figures

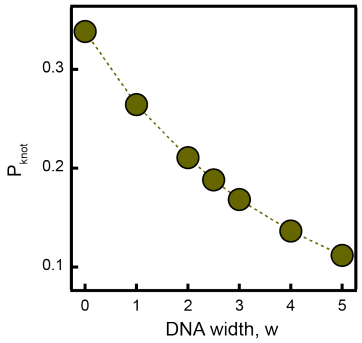

Supplementary Figure 1 Simulated total knotting probability as a function of DNA width, *w*. Total knotting probability is significantly affected by the effective width of DNA.

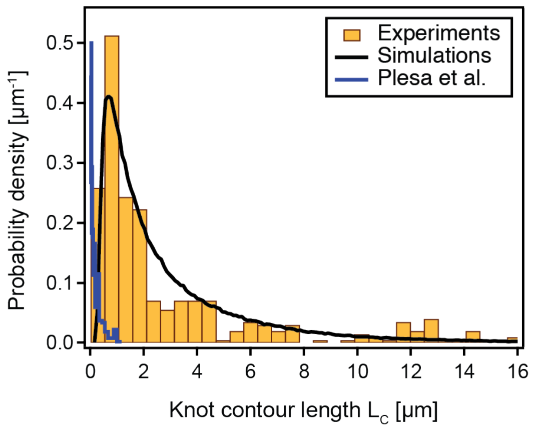

Supplementary Figure 2 Comparison of knot sizes from our experiments and simulations with the previous experiments on knots using nanopores[1].

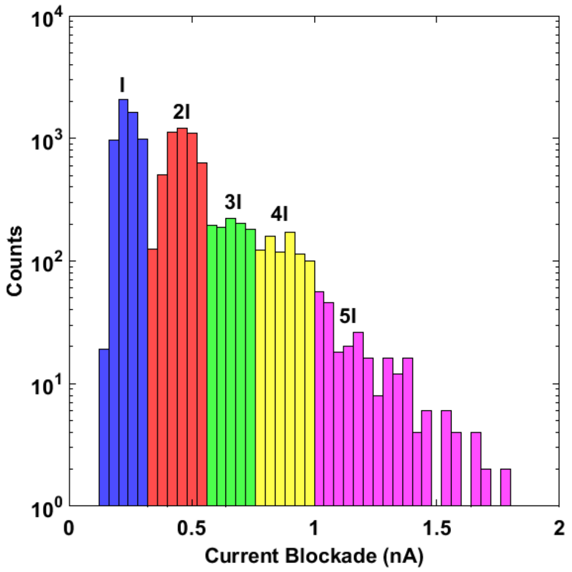

Supplementary Figure 3 Histogram showing fitted current levels from all knotted events. I and 2I correspond to one and two DNA strands passing through the pore, respectively, whereas 3I, 4I, 5I correspond to three or more strands passing through the pore simultaneously (which relates to the knotted conformations). Magenta bars include 5I and higher current drop levels which could not be clearly distinguished due to limited statistics for higher current drop levels compared to the lower current drop levels.

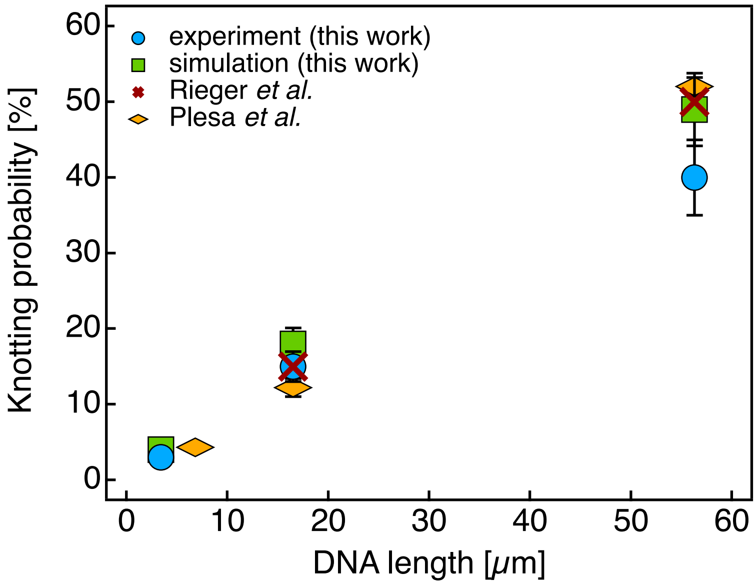

Supplementary Figure 4 Knotting probability for DNA molecules of different lengths shows good correspondence between experiments (blue circles) and simulations (green squares). Other experimental and theoretical values from the literature are shown for comparison (diamonds[1] and crosses[2]), and they show similarity to our results. All error bars show the statistical error, assuming Poisson statistics.

Supplementary References

1. Plesa, C. *et al.* Direct observation of DNA knots using a solid-state nanopore. *Nat. Nanotechnol.* **11,** 1093 (2016).
2. Rieger, F. C. & Virnau, P. A Monte Carlo Study of Knots in Long Double-Stranded DNA Chains. *PLoS Comput. Biol.* **12,** e1005029 (2016).
